# Insulin resistance and decreased spexin in Indian Patients with Type 2 Diabetes Mellitus

**DOI:** 10.6026/97320630017790

**Published:** 2021-09-30

**Authors:** G Tejaswi, CD Dayanand, K Prabhakar

**Affiliations:** 1 Department of Biochemistry, Sri Devaraj Urs Academy of Higher Education & Research, Kolar, Karnataka, India; 2 Department of General Medicine, Sri Devaraj Urs Academy of Higher Education & Research, Kolar, Karnataka, India

**Keywords:** Cardiovascular disease, type 2 Diabetes Mellitus, Spexin

## Abstract

Spexin is novel biomarker, which plays a potential role in glucose and lipid metabolisms. However, there was paucity of serum spexin levels in obesity and diabetes mellitus subjects. Hence the current study was aimed to find the relationship between the serum spexin levels in type 2 Diabetes mellitus (type 2 DM) with extrapolation of cardiovascular disease (CVD) risk. A cross-sectional study included 330 participants, subdivided as control (n=110), type 2 DM (n=110) and type 2 DM with CVD groups (n=110). HbA1c, insulin, lipid profile, spexin & leptin including blood pressure and body mass index were analyzed from all the participants. The serum spexin levels (ng/ml) were significantly decreased in type 2 DM (mean ± sd: 0.65 ± 0.03) and type 2 DM with CVD (0.48 ± 0.02) groups compared to the control (0.79 ± 0.03) group (p<0.001). The decreased spexin levels were observed in type 2 DM, and further more decreased in type 2 DM with CVD patients compared to controls indicating that spexin levels could be served as an early prediction of obesity-induced T2DM with CVD risk.

## Background:

Obesity manifests metabolically with excess body weight in adults that greatly increases the risk of type 2 diabetes Mellitus (type 2 DM) with cardiovascular disease (CVD), certain cancers, and mortality [1]. High Body Mass Index (BMI) is multi-factorial complex condition linked to genetic, epigenetic, and metabolic deregulation prone towards diabetic and cardiovascular complications [2]. Insulin resistance is recognized as a risk factor in the etiology of diseases state [3,4]. Spexin is a neuropeptide contains 14 amino acids with molecular weight 1619.9 KDa [5]. Nascent spexin on post-translational modification of α-amidation at Carboxy terminal, secretes into extracellular space is mature peptide having highly conserved sequence [6]. Physiological functions confined are inducing gastric intestinal contractions, postnatal hypoxia response, nociceptive response, inhibiting adrenal proliferation, fatty acid absorption, weight regulation, cardiovascular and renal modulation [7,8, 9,10,11,12]. Spexin binding to GalR2/GalR3 receptors belongs to G-protein family functions through the second messenger system [13]. Spexin involves regulating food intake, body weight, and energy homeostasis through neuroendocrine functions and also, it regulates glucose and lipid metabolism [14]. Spexin is also involved in Noonan syndrome- an autosomal dominant genetic disorder characterized by craniofacial abnormalities and Bjornstad syndrome rare disorder with abnormal neural deafness [7,13]. It was reported that downregulation of spexin gene and its concentration in serum is associated with obesity, type 2 DM, hypertension & CVD in metabolic syndrome conditions [15]. Leptin is having 167 amino acid residues with molecular weight of 16 kDa secreted by white adipocytes into the circulation [16]. Circulating leptin binds hypothalamus and activates the down-streaming signaling pathway to inhibit feeding behavior and accelerate energy expenditure [17,18]. In addition the endocrine function of leptin regulates the immune and inflammatory response, bone homeostasis, angiogenesis, hemeopoises, reproduction, and wound healing [19,20]. Adipose tissue mass and leptin concentration are directly proportional; therefore hyper-leptinemia is linked to obesity [18]. The increased leptin triggers an inflammatory response and promotes endothelial dysfunction by oxidative stress, which further more leads to atherosclerosis and thrombosis [19]. Mutation of the leptin gene may results in development of obesity and type 2 DM [20]. Even though, spexin is known in Type 2 DM, its involvement with insulin resistance and dyslipidemia with respect to cardiovascular complications needs to be established. Since metabolic syndrome is a higher risk for developing cardiovascular morbidity and mortality. Therefore, it is of interest to evaluate serum spexin levels correlation with LDL/HDL-cholesterol ratio and plasma insulin levels in type 2 DM, and type 2 DM with CVD complications. This research gap has become the need for the study.

## Materials & Methods:

The present cross-sectional study was conducted in the department of Biochemistry in collaboration with the department of General Medicine attached to R L Jalappa Hospital and Research Centre, of Sri Devaraj Urs Academy of Higher Education and Research, Kolar, Karnataka, India, 563103. The ethical clearance approval granted by Institutional Ethics Committee (Ref. No.: SDUMC/KLR/IEC/28/2019-20). Every participant was informed about the study and obtained written informed consent during their visit to hospital and recorded. Total of 330 subjects in the age group of 40-60years comprising both genders were sub-divided into 3 groups such as group I (G1): healthy subjects (n=110), group II (G2): type 2 DM (n=110), and group III (G3):type 2 DM withCVD (n=110) subjects. The study participants were recruited according to the inclusion and exclusion criteria.

### Inclusion criteria:

Subjects with clinically proven type 2 DM with or without hypertension and also cardiovascular complications like hypertension, Ischemic heart disease, Angina, myocardial infarction, coronary artery disease, strokes were included in the study. The criteria for diagnosis of type 2 DM as per the American Diabetic Association 2018 was considered with FBS ≥ 126 mg/dl, PPBS ≥ 200mg/dl, and HbAlc ≥ 6.5% [21].

### Exclusion criteria:

Congenital heart diseases, primary myocardial myopathies like cardiomyopathy myocarditis, Cor-pulmonale diseases, pericardial disorders, secondary diabetes (any pancreatic diseases, thyroid diseases), pregnancy & lactation, history of surgical removal of gall bladder, CNS disorders, cancer, and immune disorders were considered.

### Sample collection:

The blood sample was collected under medical supervision in aseptic conditions. Total 3ml of fasting venous blood sample were drawn from the antecubital vein using vacutainer from all study participants and transferred to plain & EDTA tubes. The plain tubes were allowed to coagulate for 20 minutes at room temperature. The plain and EDTA tubes were centrifuged at 3000 rpm for 10mins to obtain a clear serum and plasma samples respectively and stored at -80°C until analysis.

### Measurement of biochemical Parameters:

The serum lipid parameters such as total cholesterol; triglyceride, low-density lipoprotein-cholesterol (LDL-C), and high-density lipoprotein-cholesterol (HDL-C) were measured using Vitros 250 dry chemistry analyzer (Johnson & Johnson USA). As per the provided commercial kit procedure, total cholesterol was measured by cholesterol oxidase method; triglyceride by lipase hydrolysis method, and HDL-C was estimated by cholesterol ester hydrolase assay method by Vitros250 dry chemistry analyzer (Johnson & Johnson USA) and very-low-density lipoprotein (VLDL) and LDL by friedewald's formula [22]. Insulin quantification was done by enzyme immunoassay method/chemiluminescence, glycated hemoglobin HbA1c was measured by HPLC method (Biorad D10).

### Measurement of serum spexin and leptin levels:

The serum spexin & leptin levels were measured by using ELISA kits (cat: KBH3507 and L – 4260, Krishgen biosystems India) as per the protocol of commercial kit manufacturer instructions. The serum spexin and leptin levels were calculated from the obtained standard curve and the levels were expressed as ng/ml.

### Statistical analysis:

The obtained data was represented as mean ± SD/SEM. One-way analysis of variance with post hoc Bonferroni's analysis was used to find the difference between more than two groups. Pearson correlation(r) is used to correlate between the parameters. Receiver operating characteristics (ROC) curve analysis was done to assess the diagnostic feasibility of a spexin parameter in the study. The p<0.05 was considered statistically significance. All the statistical analysis was performed using SPSS software, version 20 (IBM, USA).

## Results and Discussion:

The study results and demographic data tabulated in Table 1 (see PDF). Accordingly, the age of the controls group was a 45.52 ± 10.03 (Mean ± SD) year, type 2 DM 52.23 ± 7.35 and type 2 DM with CVD subjects were, 53.51 ± 7.05. The obtained BMI for control was 17.68 ± 2.82 (mean ± SD), type 2 DM 20.17 ±2. 75, type 2 DM with CVD subjects 22.12±3.81 with level of significance between groups was p<0.001. The measured systolic blood pressure (SBP)(mmHg) levels for control type 2 DM and type 2 DM with CVD 114.61 ± 7.74 (mean ± SD), 136.55 ± 16.96151.15 ± 12.37 respectively. Similarly, the measured diastolic blood pressure (DBP) for control 80.5±1.2, type 2 DM 82.6 ± 2.9 and type 2 DM with CVD was 88.9 ± 7.1. The analysis variance showed that significant increase of SBP and DBP levels in type 2 DM and type 2 DM with CVD groups compared to control group (p<0.001). The obtained glycated hemoglobin (HbA1c) levels (%) for control found that 5.28±0.05(mean ± sem), type 2 DM 8.46±0.57, and type 2 DM with CVD as 8.76±0.18. The level of significance between the groups was found p<0.001 for theHbA1c. Plasma insulin levels (mIU/L) found in control (10.67±8.95), in type 2 DM (17.22 ± 17.62), and type 2 DM with CVD (15.25±14.53) with the level of significance between groups was p=0.002. viz., total cholesterol, triglycerides, HDL-cholesterol, LDL-cholesterol levels, and very-low-density lipoprotein (VLDL) levels were quantified and the results obtained were represented as the mean ± SD. The obtained serum lipid profile levels such astotal cholesterol, Triglycerides, HDL-Cholesterol, and LDL-Cholesterol (mg/dl) in controls group found to be 158.21±34.41, 119.19±30.265,40.72±10.30, 93.65 ± 30.49, 23.84±6.05 respectively in type 2 DM group found to be 239.64 ± 42.07, 245.53 ± 106.88, 36.52±10.61,154.01±38.57, 49.11±21.34 respectively whereas type 2 DM with CVD group were 192.82 ± 48.70, 242.75 ± 80.7, 32.5±8.73,113.02±42.62 and48.55±16.14 respectively. The level of significance was p<0.001 among the control, type 2 DM and type 2 DM with CVD groups for all the lipid profile parameters. According to the results, total cholesterol, triglycerides, VLDL, and LDL-C values increased and HDL values decreased intype 2 DM and type 2 DM with CVD patients than controls. Serum spexin (ng/ml) levels obtained from control 0.80±0.38 (mean ± sem), type 2 DM 0.64±0.32andtype 2 DM with CVD was0.49±0.25.In present study, circulating spexin levels were significantly decreased in type 2 DM group (p<0.001) compared to the control. Furthermore decrease of serum spexin levels in observed in type 2 DM with CVD group compared to the control (p<0.001). In addition the serum spexin levels were significantly decreased intype 2 DM with CVD group compared to type 2 DM group (p<0.001) (Figure 1 - see PDF) (Table 1 - see PDF).

Pearson correlation analysis was performed to determine the correlation between the serum spexin and different cardio metabolic parameters. The negative correlation was observed between the serum spexin levels and triglycerides (r=-0.068,p= 0.48), DBP (r= -0.092, p= 0.342), BMI (r= -0.046, p= 0.62), and W/H ratio (r=-0.086, p=0.372) in type 2 DM group. In contrast the positive correlation was observed between the serum spexin levels and insulin (r=0.1, p=0.2), total Cholesterol(r=0.4, p=0.009), HDL-C(r=0.02, p=0.0.7), LDL-C(r=0.3, p=0.001), LDL/HDL ratio(r=0.09, p=0.3), SBP(r=0.09, p=0.3) in type 2 DM. In addition we observed the significant positive correlation between the serum spexin levels and insulin (r= 0.24, p=0.009), triglycerides (r= 0.23*, p=0.01), DBP (r=0.055,p=0.05) and there was significant negative correlation between the serum spexin and total cholesterol (r=-0.08,p= 0.38), HDL (r=-0.07,p= 0.48), LDL r=-0.15,p= 0.11), SBP (r= -0.06, p= 0.5), BMI (r= -0.11, p= 0.2), and W/H ratio (r=-0.05, p=0.5) in type 2 DM with CVD group (Table 2,3 & 4 - see PDF). The obtained serum leptin levels (ng/ml) for the controls23.32±1.09, type 2 DM 28.83 ± 0.62and in type 2 DM with CVD 35.72±0.98 groups (Table 1 - see PDF) (Figure 2 - see PDF). The leptin levels were significantly increased in type 2 DM and type 2 DM with CVD groups compared to the control. We also observed that the serum leptin levels were increased significantly in type 2 DM with CVD group compared to type 2 DM (p<0.001).

The obtained spexin/leptin ratio in the control group was 1:29, type 2 DM 1:45, and type 2 DM with CVD 1:74. In other view spexin/leptin ratio for the control, type 2 DM and type 2 DM with CVD was 56.9±7.7, 23.82±1.25, and 14.81±0.58 respectively. The spexin/leptin ratio was significantly decreased in type 2 DM and type 2 DM with CVD groups compared to the control group. We also observed that the spexin/leptin ratio were decreased significantly in type 2 DM with CVD group compared to type 2 DM (p<0.001) (Table 1 - see PDF) (Figure 3 - see PDF). The obtained LDL-C/HDL-C ratio levels were control 2.44±1.06, type 2 DM 4.81±2.71, and type 2 DM with CVD 3.72±1.82 groups. The LDL-C/HDL-C ratio significantly higher in type 2 DM, and in type 2 DM with CVD groups compared to control (p<0.001) (Table 2, 3 & 4 - see PDF) (Figure 4 - see PDF). ROC curve analysis for significant parameters was depicted. The data shows the sensitivity & specificity of the Spexin as a marker in type 2 DM with CVD when compared to controls in type 2 DM. The area under the ROC curve was 0.83 for Spexin levels in type 2 DM with CVD (p<0.001***) and which has a cutoff value of [0.51ng/ml], the sensitivity and specificity of Spexin were [81%] and [62%], respectively whereas AUC in type 2 DM was 0.65 for Spexin and which has cutoff value of [0.62ng/ml], the sensitivity and specificity of Spexin were[65%] and [62%], respectively. as shown in (Figure 5 & 6 - see PDF). The current study reported that serum leptin levels were increased in type 2 DM patients and type 2 DM with CVD patients than controls. Additionally leptin showed a correlation (positive/negative) with cardio metabolic parameters (shown in results) (Table 2,3 & 4 - see PDF) in type 2 DM or type 2 DM with CVD subjects. It was reported that there was link between high leptin levels and increased risk of cardiovascular disease in type 2 DM patients. Ridulescu A et al., (2020) reported that an increased risk of incident type 2 DM with increasing leptin levels [23]. In line with the reported studies, the current study results shown the increased leptin levels in type 2 DM and type 2 DM with CVD patients indicating that leptin is known biomarker in obesity, type 2 DM with CVD patients.

The current study also reported that circulating spexin levels were observed as low in type 2 DM patients and type 2 DM with CVD patients than healthy controls. Additionally spexin showed a correlation (positive/negative) with cardio metabolic parameters (shown in results) in type 2 DM and type 2 DM with CVD patients. Studies in humans on spexin are very sparse. Several studies reported that a low level of circulating spexin in type 2 DM patients and it showed negatively correlation with blood glucose, HbA1c, TG/LDL/TC [11,24,25,26]. In contrast studies were reported that spexin levels were increased in women with gestational diabetes mellitus [27,28,29]. Lin CY et al., 2018 reported that spexin has a role in bile acid synthesis regulation, lipid metabolism and concluded that impaired spexin activation leads to accumulation ofcholesterol [30]. Nevertheless, Hodges et al., (2018) reported that there was decreasing of spexin levels in obesity or type 2DM in adults [31]. Kolodziejski PA et al., (2021) reported that spexin treatment for 30 days in obese and diabetic mice regulates hormonal and metabolic status, improves insulin sensitivity, improves glucose tolerance and reduces body weight [32]. Khadir A et al., (2020) also reported that regular physical exercise can also increase the levels of spexin to manage obesity and healthy lifestyle [33].In line with the reported studies, the current study results shown the decreased spexin levels in type 2 DM and type 2 DM with CVD patients indicating that spexin may have potential role in obesity induced diabetes. Hence, the spexin may also consider as biomarker in obese and type 2 DM subjects along with the leptin. However, longitudinal prospective studies are needed to know the spexin role in obesity and type 2 DM as biomarker.

The availability of information on the Spexin/leptin ratio and its correlation with diseases state is limited. In the majority, study reports are confined to either independent spexin or leptin. Kumar S etal., (2018) reported an inverse association between spexin and leptin in adolescents with obesity [14]. Hence in the current study scenario, we attempted to evaluate the spexin/leptinratio in type 2 DM and with CVD risks.In our study results we observed that the spexin/leptin ratio was progressively decreased in type 2 DM and type 2 DM with CVD groups compared to control. As Kumar S etal., (2018) suggested inverse association between spexin and leptin in adolescents with obesity, the current study results also indicating that spexin/leptin ratio could be helps for diagnosis, prognosis, and intervention monitoring of CVD risks in type 2DM patients. Therefore, measurement of spexin level regarded as a potential biomarker to represent obesity, type 2 DM, and cardio-metabolic complications. Spexin and insulin concentration in the control group is in a balanced state, however, altered levels are observed in disease states. Hence, the results of the study spexin/leptin ratio associated with atherogenic potency as denoted by elevated LDL/HDL-C ratio. Therefore the study results should be interpreted cautiously that spexin /leptin serves as an early marker of metabolic syndrome particularly the onset of obesity, type 2 DM and type 2 DM with CVD.

## Limitations of the study:

The genetic analysis of Spexin gene associated with low levels of circulating Spexin has become the limitation.

## Conclusion:

Data shows that decrease spexin levels were observed in type 2 diabetes mellitus subjects and further remarkable reduction in type 2 DM with CVD subjects. Therefore, decreased Spexin concentration serves as an early prediction of risk involved in obesity-induced T2DM with cardiovascular complications.

## Funding:

Nil
